# Functional modelling of a novel mutation in *BBS5*

**DOI:** 10.1186/2046-2530-3-3

**Published:** 2014-02-21

**Authors:** Mohamed H Al-Hamed, Charles van Lennep, Ann Marie Hynes, Paul Chrystal, Lorraine Eley, Fatimah Al-Fadhly, Riham El Sayed, Roslyn J Simms, Brian Meyer, John A Sayer

**Affiliations:** 1 International Centre for Life, Institute of Genetic Medicine, Newcastle University, Central Parkway, Newcastle NE1 3BZ, UK; 2 Genetics Department King Faisal Specialist Hospital and Research Centre, PO Box 3354, Riyadh 11211, Saudi Arabia; 3 Department of Paediatrics, Maternity & Children’s Hospital, PO Box 6205, Al Madina, Al Munawara, Saudi Arabia; 4 Department of Clinical and Chemical Pathology, Kasr Al-Ainy Faculty of Medicine, Cairo University, Cairo, Egypt

**Keywords:** BBS, Zebrafish, Pronephros, Cilia, Situs inversus, Retinopathy

## Abstract

**Background:**

Bardet-Biedl syndrome (BBS) is an autosomal recessive ciliopathy disorder with 18 known causative genes (*BBS1-18*). The primary clinical features are renal abnormalities, rod-cone dystrophy, post-axial polydactyly, learning difficulties, obesity and male hypogonadism.

**Results:**

We describe the clinical phenotype in three Saudi siblings in whom we have identified a novel mutation in exon 12 of *BBS5* (c.966dupT; p.Ala323CysfsX57). This single nucleotide duplication creates a frame shift results in a predicted elongated peptide. Translation blocking Morpholino oligonucleotides were used to create zebrafish *bbs5* morphants. Morphants displayed retinal layering defects, abnormal cardiac looping and dilated, cystic pronephric ducts with reduced cilia expression. Morphants also displayed significantly reduced dextran clearance via the pronephros compared to wildtype embryos, suggesting reduced renal function in morphants. The eye, kidney and heart defects reported in morphant zebrafish resemble the human phenotype of *BBS5* mutations. The pathogenicity of the novel *BBS5* mutation was determined. Mutant mRNA was unable to rescue pleiotropic phenotypes of *bbs5* morphant zebrafish and in cell culture we demonstrate a mislocalisation of mutant BBS5 protein which fails to localise discretely with the basal body.

**Conclusions:**

We conclude that this novel *BBS5* mutation has a deleterious function that accounts for the multisystem ciliopathy phenotype seen in affected human patients.

## Background

BBS is a rare autosomal recessive disorder [1] which is now considered, given its pleiotropic and multisystem features and the known function of BBS proteins, to be a ciliopathy. The incidence of BBS, has been reported to be 1 in 125,000 in the UK [1] and 1 in 160,000 in Switzerland [2]. It is more common in some small populations such as Newfoundland (1 in 18,000) [3] and the Bedouin community of Kuwait (1 in 13,500) [4] where there may be a founder effect [5] and an increased incidence of consanguineous relationships [6].

Following a comprehensive analysis of known BBS patients in the UK, Beales *et al*. provided revised diagnostic criteria for the clinical syndrome. Currently, four out of six primary features of BBS or three out of six primary features and at least two of eleven secondary features are required for a clinical diagnosis [7] The primary features include renal abnormalities (renal cystic dysplasia and anatomical malformations), rod-cone dystrophy, post-axial polydactyly, learning difficulties, truncal obesity and male hypogonadism [7]. The renal manifestations of BBS are the most life-limiting component of the disorder [8,9]. Out of the 11 secondary features, four (brachydactyly, developmental delay, speech deficit and high- arched palate) have been reported to affect over 40% of BBS patients [7]. There is considerable variation in the phenotypes of affected individuals, even between affected siblings [10]. Additional phenotypes have also been reported in BBS patients, including cardiac malformations [11], situs inversus [12] and anosmia [13].

BBS is a genetically heterogeneous disorder with mutations in 18 known genes (*BBS1-18*) [14-16] accounting for approximately 80% of patients with BBS. In 2004, Li *et al*. identified *BBS5* using an elegant comparative genomics method [17]. Comprehensive bioinformatics carried out on BBS5 by Nachury *et al*. discovered that two novel domains in the BBS5 protein were similar to pleckstrin homology (PH) domains which bind to phosphoinositides [18]. Phosphoinositides are phosphorylated forms of phosphatidylinositol that are found in cell membranes and have functional links with RABs including Rab8 [19]. BBS5 isolates bind to phosphoinositides *in vitro* and because both siRNA inhibition of *BBS5* and inhibition of phosphoinositides resulted in loss of ciliation [18], this suggests that BBS5 binding to phosphoinositides is needed for ciliogenesis [18].

We report an interesting novel *BBS5* mutation in a consanguineous family from Saudi Arabia. Given that this mutation was not a missense or nonsense mutation but led to a predicted elongated transcript that may retain functional activity, we sought to characterise this mutation further. The pathogenicity of this novel mutation was assessed in a zebrafish model, where it was unable to rescue pleiotropic phenotypes of *bbs5* knockdown and in cell culture assays, where the novel mutation led to BBS5 mutant protein mislocalisation in renal epithelial cells (Qiagen, Manchester, UK).

## Methods

### Study cohort

This study has been approved by the research advisory council of King Faisal Specialist Hospital, Riyadh, Saudi Arabia (RAC#2050 045). Following informed consent, DNA was extracted from peripheral blood cells using the Gentra Systems Puregene DNA Isolation kit (Qiagen, Manchester, UK).

### Homozygosity mapping and mutation analysis

To search for homozygous regions and possible chromosomal abnormalities, all available family members were genotyped with an Affymetrix® CytoScan™ Array. The primary data was analysed using Chromosome Analysis Suite (ChAS) software (Affymetrix). Direct sequencing of all coding exons and exon-intron boundaries of *BBS5* was performed. Primer sequences are available upon request. PCR products were sequenced using BigDye™ Terminator Cycle Sequencing kit (PE Applied Biosystems, Bedford, MA, USA). Sequences were analysed using Mutation Surveyor® software Version 3.24 (SoftGenetics LLC, State College, PA 16803, USA). Mutations were labelled according to the Human Genome Variation Society (HGVS) recommendations version 2.0. A control DNA panel from 96 individuals from a Saudi Arabian population was used to screen for the novel sequence variant.

### Zebrafish studies

For zebrafish studies, all procedures were performed under Home Office UK license regulations. Transgenic fluorescent reporter fish were used for studying cardiac morphology (*cmlc2*:GFP, the GFP gene under the control of the cardiac myosin light chain 2 gene promoter [20]) and renal morphology (*cldnb:lynGFP*, a membrane-localised variant of the GFP gene under the control of the *claudin b* gene promoter [21]). Other zebrafish lines used were wildtype (WT) AB and *golden*. Zygotes were collected from natural spawning and placed in petri dishes of E3 medium (5 mM NaCl, 0.17 mM KCl, 0.33 mM CaCl_2_, 0.33 mM MgSO_4_) [22].

Morpholino oliogonucleotides (MOs) were manufactured by Gene Tools LLC (Philomath, OR, USA). The standard control (5′-CCTCTTACCTCAGTTACAATTTATA-3′) and *p53* translation targeting (5′-GCGCCATTGCTTTGCAAGAATTG-3′) MO sequences were of pre-established design. MO sequences for *bbs5* translation targeting (ATG) (5′-GATCACTGTCTGCGTATATTGTCGA-3′) were designed with reference to the zebrafish genome assembly Zv9.

Stock MOs in RNase free water were diluted with 0.05% phenol red in Danieau buffer to produce the solution for injection. Dilutions of 3 to 12 ng of MO per 2 nl of solution were used. All MOs, except the *p53* ATG MO, were used individually. The *p53* ATG MO was co-injected in a 1:1 mixture with the *bbs5* ATG MO in order to assess any p53-mediated off-target effects [23] of the *bbs5* ATG MO. MOs were microinjected under light microscopy into the yolk of one- to four-cell embryos, using a glass micropipette and an Eppendorf FemtoJet pneumatic micro-injector. The micropipettes were calibrated by measuring droplet size on a reticle so that 2 nl of MO solution were injected into each embryo. After microinjection, the embryos were placed in fresh E3 medium, approximately 50 embryos per petri dish, and incubated at 28.5°C. Mortality counts were performed at 3 and 24 hours post fertilisation (hpf) on both injected and uninjected control embryos. Clutches where the mortality rate in uninjected embryos was above 50% at 24 hpf were discarded.

Human *BBS5* EST clone (BC044593.1) inserted into vector pBluescriptR was obtained (GeneService) and the clone was sequenced to verify clone length and fidelity. To obtain mutant *BBS5* (c.966dupT), site directed mutagenesis was employed. Using a T7 forward oligonucleotide primer and reverse oligonucleotide primer within the 3′UTR of *BBS5*, PCR products of 1,194 bp were obtained and used as a template to perform an *in vitro* synthesis of large cRNA (mMessage mMachine T7 kit (Ambion, Inc., AM1340)). cRNA samples were purified by phenol:chloroform extraction and isopropanol precipitation.

cRNA was mixed 1:1 with *bbs5* ATG MO in its microinjection solution to achieve 100 pg of mRNA and 5 ng of MO in 2 nl of the final solution. This solution was then microinjected into one- to four-cell embryos as described. Western blotting was used to confirm WT BBS5-NT-GFP and c.966dupT mutant BBS5*-*NT-GFP protein expression. Briefly, protein was extracted using an extraction buffer (4 M urea, 125 mM Tris pH 6.8, 4% SDS, 10% glycerol, 5% beta-mercaptoethanol, and 0.02% bromophenol blue). Samples were separated using SDS-polyacrylamide gel electrophoresis and then electrophoretically transferred to Hybond-C-extra nitrocellulose membranes (Fisher Scientific UK Ltd). Protein was detected using an anti GFP-HRP (Santa Cruz sc-9996, Santa Cruz Biotechnologies Inc., Santa Cruz, CA, USA) (1 in 5,000 dilution) for one hour at room temperature before developing and imaging.

### Zebrafish phenotypic data

Light and fluorescent microscopy was performed to monitor the embryos at 24, 48 and 72 hpf. Embryos were dechorinated (using forceps) and immobilised in 0.1% tricaine in E3. The phenotypes of each fish at 72 hpf were recorded, noting the presence of pericardial effusion, tail length and appearance, body oedema and venous stasis in the cardiac venous sinus. Pronephric duct morphology assessment in *cldnb:lynGFP* fish was performed at 48 hpf and 72 hpf. For ciliary staining, embryos were fixed in acetone and stained with mouse acetylated tubulin antibody (Sigma, UK), secondarily detected with Alexa Fluor 594 (Invitrogen Life Sciences Ltd.). To assess cardiac morphology (D-loop, L-loop or failure of looping and cardiac chamber appearance), 72 hpf *cmlc2*:GFP embryos were used.

Fish were classed phenotypically as i) normal if they were indistinguishable from uninjected fish; ii) mildly affected if they had small eyes, mild pericardial effusion and mild/no tail defects; iii) moderately affected if they had a large pericardial effusion and moderate tail defects (obvious shortening and large kinks/curls in their tails). Fish exhibiting a very severe or ‘monster phenotype’ [24] (no or very short malformed tail, widespread oedema, malformed eyes and minimal cardiac muscle contraction) were noted but not used in the analysis of morphology [24]. Eye size was measured using superior view photograph using the long axis length of each zebrafish eye. Images were taken with a Leica DF425C camera and the Leica Application Suite V3 program and analysis was performed using ImageJ Software (National Institutes of Health).

### Histological analysis of zebrafish morphants

Seventy-two hpf control and *bbs5* ATG MO injected (mild to moderate phenotype) embryos were euthanised in 4% tricaine and fixed in 2% glutaraldehyde in 0.1 M cacodylate buffer for two hours at room temperature. They were then dehydrated in a stepwise manner in acetone then impregnated with epoxy resin in stepwise fashion. Embryos in 100% epoxy resin were then placed individually in coffin moulds (EMS #70905-01) and polymerised at 60°C for 24 hours. One micrometer transverse microtome sections were stained with 1% toluidine blue in 1% borax. In parallel, zebrafish were also embedded in (Electron Microscopy Sciences, Hatfield, PA, USA) and allowed to set in a low humidity environment. A glass knife was used to cut 5 μm sections which were then stained by Lee’s trichrome and mounted in Histamount. Sections were then analysed on a Zeiss Axioplan 2 light microscope and images were taken (Zeiss AxioCam HRc camera).

### Renal function studies in zebrafish embryos and morphants

Uninjected control embryos and *bbs5* ATG MO injected embryos with a mild to moderate phenotype were selected at 48 hpf and dechorinated and immobilised using 0.1% tricaine in E3. Four nanograms of 10 kDa tetramethylrhodamine dextran (dissolved in RNase free water) were microinjected into the cardiac venous sinus, lateral and inferior to the heart. To confirm successful cardiac injection, embryos were checked for the presence of fluorescence within the heart under fluorescent microscopy. A lateral view fluorescent image of each fish was taken at 3, 24 and 48 hours post dextran injection keeping exposure, saturation, gamma, gain and zoom level were all constant. A 100 × 100 pixel square from the centre of the heart for each fish was taken and the average intensity measured using ImageJ software. Pronephric ducts and cilia were visualised using a Nikon A1R confocal microscope and Elements software package.

### Cell culture and transfection

Routine tissue culture techniques were used to facilitate the growth of the Human Embryonic Kidney 293 cells (HEK293) mammalian cell line [25]. Cell lines were cultured in DMEM. Human *BBS5* (EST clone reference 5272889) and mutant *BBS5* (c.966dupT) and WT plasmids were subcloned into pcDNA3.1-NT-GFP vector (Invitrogen Life Technologies Ltd, UK). Following subcloning into pcDNA3.1-NT-GFP, clones were fully sequenced to confirm sequence fidelity, reading frame and orientation (data not shown). Following successful cloning, large scale preparations of plasmid cDNA were obtained using overnight culture in Luria Broth and isolation and isolation of DNA using a MaxiPrep kit (Qiagen, Manchester, UK). For transfection, Lipofectamine 2000 reagent (Life Technologies, UK) was used empirically as per the manufacturer’s protocol. Cells were fixed 24 to 48 hours post transfection in 4% paraformaldehyde (PFA) and permeabilised using 0.5% Triton-X100 (Sigma, UK) in PBS solution.

### Cell immunofluorescence staining

Following blocking with 5% BSA, cells were labelled with mouse acetylated tubulin (1:1,000; Sigma, UK) and detected using Alexa Fluor 594 donkey anti-mouse (1:200; Invitrogen Life Technologies Ltd, UK). Labelling with pericentrin antibody (1:200; Abcam, Cambridge, UK) was secondarily detected using an anti-rabbit Cy3 antibody (1:200, Invitrogen Life Technologies Ltd, UK). Cells were imaged using confocal laser scanning microscopy, (Nikon U.K. Ltd).

### Statistics

Zebrafish phenotype data was analysed using chi-squared test. Eye size and cardiac fluorescence intensity were compared using an unpaired two-sample *t*-test (MiniTab 16 software, Minitab Ltd., Coventry, UK).

## Results

### Molecular genetic diagnosis

**Figure 1:**
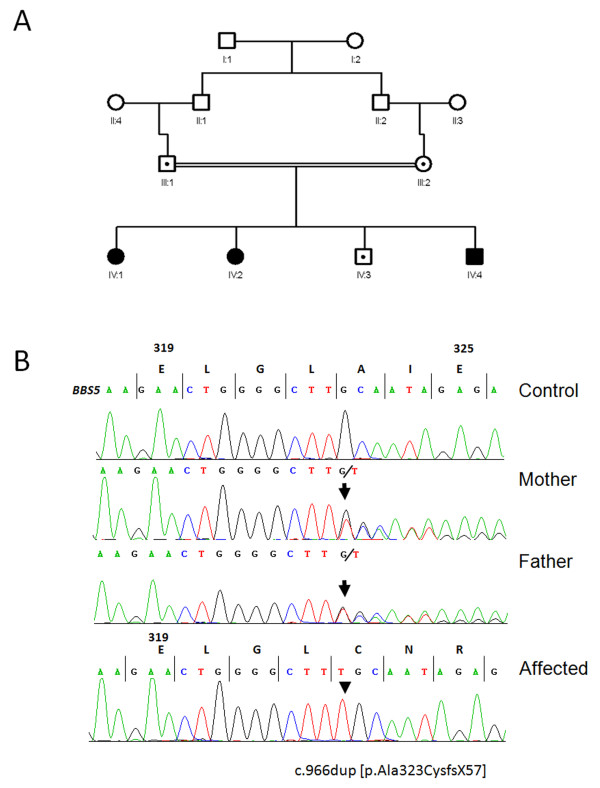
**Family structure and molecular genetic diagnosis of a novel *BBS5 *** **mutation. (A)** Pedigree diagram showing three affected siblings (shaded figures) from a consanguineous family with BBS. Circles represent females, squares represent males. Carrier (heterozygous) status is shown by a dotted symbol. **(B)** Sequencing chromatogram s from exon 12 of *BBS5* gene showing a homozygous duplication of T nucleotide in affected son (IV:4) and segregation of the mutation from each parent.

We performed homozygosity mapping using SNP markers on all three affected patients and both parents of a Saudi Arabian family (Figure 1A) where a clinical diagnosis of BBS was suspected in three of their children (Table 1). All three affected children met the current diagnostic criteria for BBS, each having 4/6 primary and 2/11 secondary BBS features.

**Table 1:** Clinical features of three affected siblings

		**Primary BBS features**						**Secondary BBS features**		
	**Age at presentation (years)**	**Post axial polydactyly**	**Retinal dystrophy**	**Renal anomalies**	**Learning difficulties**	**Hypogonadism**	**Obesity**	**Developmental delay**	**Congenital heart disease**	**Astigmatism**
Affected female	9	Y	Y^a^	Y^b^	Y	n/a	Y	Y	N	Y
Affected female	7	Y	N	Y^c^	Y	n/a	Y	Y	VSD	N
Affected male	3	Y	N	N	Y	Y	Y	Y	VSD	N

^a^night blindness and retinal dystrophy developed at aged 12.

^b^dilatation of left renal pelvis.

^c^bilateral small kidneys on USS. VSD, ventricular septal defect.

A region of homozygosity on chromosome 2 (chr2:170,310,006-170,383,165) shared by all three affected children was identified (data not shown) which included two genes: a known BBS gene, *BBS5* and *KBTBD10*. The *BBS5* gene contains 12 exons, all of which are coding and its full-length transcript is 3,475 bp and 341 amino acid residues. Direct sequencing of all 12 coding exons of *BBS5* was performed and a novel homozygous frameshift mutation c.966dupT (p.Ala323CysfsX57) in exon 12 of *BBS5* was identified in all three affected individuals, with the mutation segregating from both parents (Figure 1B). The unaffected son was heterozygous for the mutation. The mutation affects a highly conserved region of the BBS5 protein and results in a predicted elongated peptide that has 379 amino acids instead of 341 in the WT. The mutation was not found in 100 DNA normal controls from a Saudi Arabian population.

### *In vivo* modelling of novel *BBS5* mutation in zebrafish

Since our main goal was to demonstrate that the c.966dupT *BBS5* mutation is pathogenic, we wanted to assess whether the mutated BBS5 retains full function. One way of testing this is to perform *in vivo* rescue experiments and the zebrafish is a good model to use for this for a number of reasons. The zebrafish can be used to assess the pleiotropic phenotype typical of BBS, and has been used successfully to characterise phenotypes of *bbs1*, *bbs2*, *bbs4*, *bbs5*, *bbs6*, *bbs7* and *bbs8*[26,27]. The zebrafish model has also been successfully utilised to examine the effect of pair-wise BBS gene knockdown in order to study genetic interactions [27]. In addition, morphant zebrafish can be used in co-injection experiments to determine the degree of rescue of WT and mutated mRNAs.

**Figure 2:**
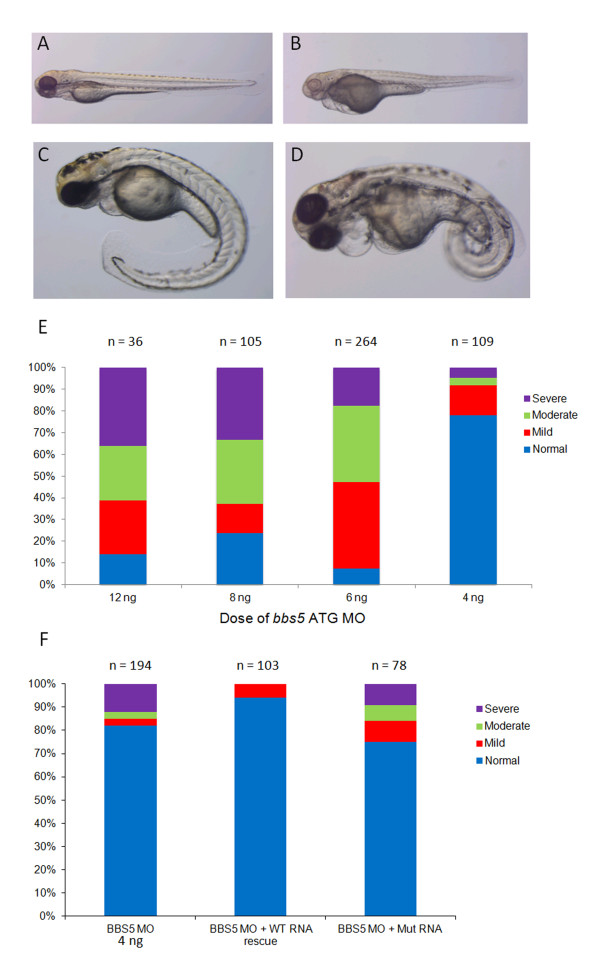
**Phenotypic spectrum following *bbs5 *** **ATG MO injection and mRNA rescue.** At 72 hpf following *bbs5* MO injected (+/- mRNA rescue) zebrafish were phenotyped under light microscopy. Representative images are shown: **(A)** wildtype (WT) phenotype; **(B)** mildly affected morphant, showing a pericardial effusion and small eyes; **(C)** moderately affected morphant with a pericardial effusion, small eyes and a curly tail and **(D)** a severely affected morphant with a large pericardial effusion, small eyes and severe tail curvature. **(E)** Dose–response analysis of phenotypes following *bbs5* ATG MO injection. Fish were classed phenotypically as normal if they were indistinguishable from uninjected fish; mildly affected if they had small eyes, mild pericardial effusion and mild/no tail defects; moderately affected if they had a large pericardial effusion and moderate tail defects, and severe if there was absent or very short malformed tail, widespread oedema, malformed eyes and minimal cardiac muscle contraction). Note a trend of increasing severity of phenotypes from 4 ng to 12 ng MO dose. The number of embryos (n) injected for each dose are shown. **(F)** Phenotypes of *bbs5* ATG MO injected fish compared to co-injected WT BBS5 mRNA and mutant (Mut) BBS5 mRNA.

Using zebrafish embryos, MOs were designed and injected to modify gene expression of *BBS5*. Delivered doses of 4 ng, 6 ng, 8 ng and 12 ng of *bbs5* ATG MO each produced a range of phenotypes at 72 hpf including normal, mild, moderate and severe (Figure 2). Mild phenotypes included pericardial oedema and mild eye and tail defects. Moderate and severe phenotypes included curly tails, small eyes and prominent cardiac and pericardial oedema. There was a dose–response progression in phenotype from mild to more severe as doses increased, consistent with specificity of the *bbs5* ATG MO (Figure 2E). Using a 4 ng dose, 22% of embryos displayed any phenotype, whilst a 12 ng dose produced a mild phenotype in 25%, a moderate phenotype in 25% and a severe phenotype in 36% of embryos. A dose of 6 ng *bbs5* ATG MO was used for subsequent phenotyping experiments. To exclude off-target effects of *bbs5* MO injection, embryos were co-injected with 6 ng of both *p53* and *bbs5* ATG MOs (n = 144) or 6 ng *bbs5* ATG MO alone (n = 143). There was no detected difference in phenotype between the two groups and they both had similar distributions of phenotypic severity (data not shown).

### Retinal phenotype

**Figure 3:**
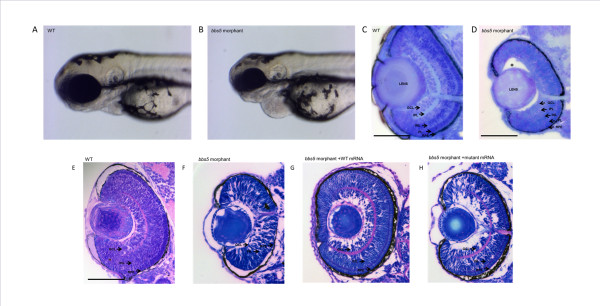
**Microphthalmia and retinal layering defect in *bbs5 *** **morphants and phenotypic rescue with mRNA co-injection.** Light microscopy of 72 hpf embryos show a reduction in eye size in between **(A)** wildtype (WT) and **(B)***bbs5* morphants. Retinal sections demonstrating **(C)** WT retina with normal, well demarcated retinal layering compared to **(D)** morphant retina displaying partial loss of retinal layering, loss of photoreceptor layer and separation of lens from retina (*). In mRNA rescue experiments, WT mRNA or mutant mRNA was co-injected with *bbs5* MO. **(E)** Retinal layers are preserved in WT and **(F)** disrupted in *bbs5* morphants. **(G)** WT mRNA is able to rescue the retinal phenotype and eye size whilst in the **(H)** mutant mRNA phenotype retinal layers remain disrupted and the eye size small. Scale bar 100 um. GCL, ganglion cell layer; INL, inner nuclear layer; IPL, inner plexiform layer; PL, photoreceptor layer, RPE, retinal pigment epithelia.

Given that the 72 hpf phenotype of morphants under light microscopy included microphthalmia (Figure 3B), we attempted to quantify this further. The mean eye diameter of *bbs5* ATG morphant fish with a mild to moderate phenotype was significantly smaller (243 pixels (n = 30)) compared to control fish (328 pixels (n = 28), *P* < 0.001). Histological examination of WT embryo retina at 72 hpf displayed normal retinal layering, with each layer appearing intact and distinct (Figure 3C). In *bbs5* morphants, retinal sections demonstrated a microphthalmia with partial loss of the retinal layering. The photoreceptor layer was disrupted when compared to WT, and the lens and retina seemed to have separated from each other in the morphant embryos (Figure 3D).

### Cardiac phenotype

**Figure 4:**
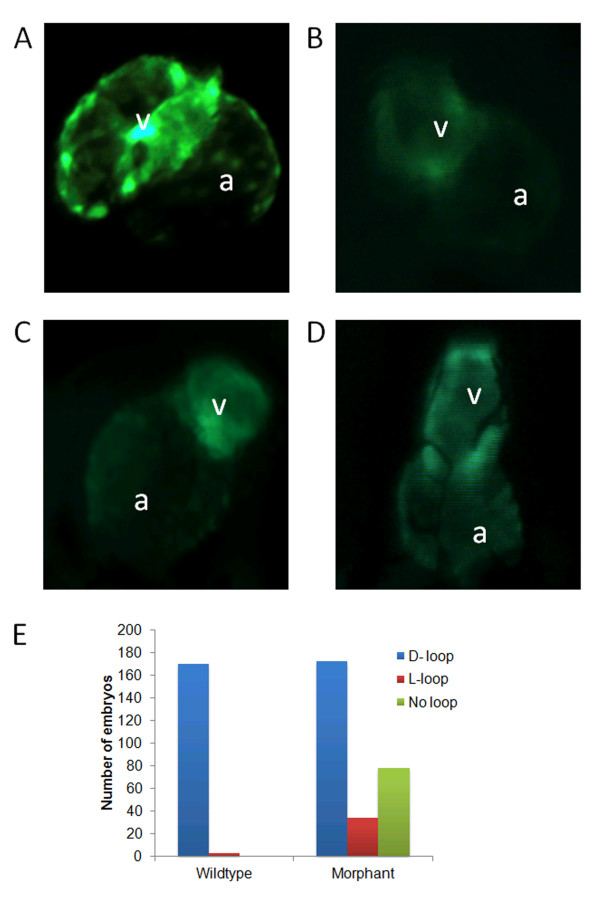
***bbs5 *** **morphants display cardiac anomalies including *situs inversus*.** Wildtype (WT) and morphant *cmlc2:GFP* embryos were imaged at 48 hpf. **(A)** WT heart showing normal looping (D-looping) with the atrium (a) on the left hand side of the embryo and the ventricle (v) on the right. **(B)** Morphant heart with preserved D-looping. **(C)** Morphant heart with looping in the opposite direction (L-looping) resulting in the atrium on the right of the embryo and the ventricle on the left. **(D)** Morphant heart, which shows a failure of looping, resulting in a heart with neither the atrium nor ventricle leaving the midline. **(E)** 98% of WT embryos displayed D-looping whilst just 61% of morphant embryos exhibited D-looping, 12% exhibited L-looping, and 27% of morphant hearts were unlooped (*P* < 0.001).

At 48 hpf *bbs5* ATG MO injected morphant embryos displayed abnormal hearts (Figure 4B-D). In 98% of WT embryos (n = 173), the hearts had a single atrium and ventricle that had undergone D-looping, resulting in the atrium being on the left side of the embryo and the ventricle on the right (Figure 4A). Morphant embryos (n = 218) demonstrated L-looping in 12%, resulting in the atrium being on the right side of the embryo and the ventricle on the left. A larger proportion of morphant hearts failed to loop (27%), resulting in both the atrium and the ventricle remaining in the medial position (Figure 4E).

### Renal phenotype

**Figure 5:**
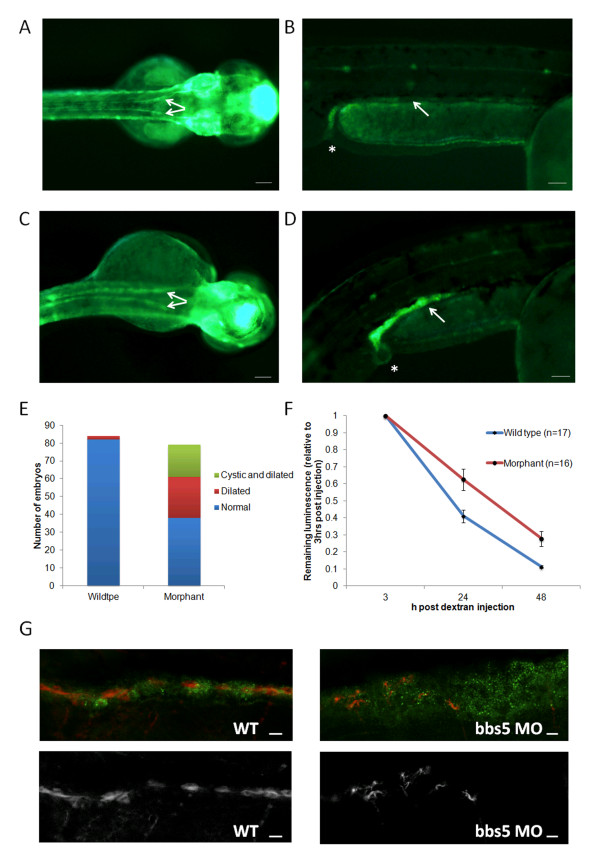
**Characterisation of pronephros structure and function in *bbs5 *** **morphants. (A-D)** Superior and lateral views of a *cldnb*:*lynGFP* embryo displaying **(A,B)** normal pronephric ducts (arrowed) and cloaca (*) in wildtype (WT) zebrafish. **(C,D)***bbs5* morphant embryos displayed dilated and tortuous pronephric ducts (identified by GFP fluorescence) and a **(D)** cloacal cystic dilatation. Scale bars are 100 μm. **(E)** Quantification of pronephros abnormalities in WT and *bbs5* morphant embryos. **(F)** Estimation of GFR in zebrafish embryos was performed by measuring change in cardiac luminosity of both WT and morphant embryos after cardiac sinus fluorescent dextran injection. Mean luminescence +/- SEM (arbitrary units) is plotted versus time, up to 48 hours post injection. Morphant embryos retained significantly more fluorescent dextran at 24 hours (*P* = 0.005) and at 48 hours (*P* = 0.002). **(G)** At 72 hpf, compared to WT control, *bbs5* morphant embryos revealed disrupted and fewer numbers of cilia in the dilated pronephros. Scale bar 10 μm).

Pronephric duct morphology was analysed in *cldnb:lynGFP* embryos, which have a fluorescent pronephric duct (Figure 5A-D and Additional file 1: Figure S1). Examination of pronephric ducts under light microscopy is very subjective but examining embryos with fluorescent pronephric ducts allowed accurate phenotyping to be performed in living fish (Additional file 1: Figure S1). Dilated pronephric ducts were observed in 2% of WT embryos (n = 84), compared to 29% of morphant embryos (n = 79). In addition, 23% of morphant embryos displayed pronephric or cloacal cysts. No cysts were observed in WT embryos (Figure 5E). Using WT mRNA we were able to rescue the cystic phenotype. In addition, we utilised zebrafish embryos to determine evidence of kidney dysfunction, using methodology initially developed by Hentschel *et al*. [28]. Embryonic renal clearance in morphant zebrafish may be assessed by microinjection then subsequent tracking the elimination of a fluorescently-tagged dextran through serial image capture [29]. To estimate renal function *bbs5* morphants (n = 16) and WT (n = 17) embryos were followed over a 48-hour period after cardiac venous sinus fluorescent dextran injection. Two morphant embryos and one WT embryo died before the end of the observation period and were excluded from the analysis. At 24 hours post dextran injection, morphant embryos retained significantly more fluorescence than WT embryos. The mean 24-hour post injection to 3 hours post injection fluorescence ratio was 0.630 for morphants, compared to 0.417 for WT embryos (*P* = 0.005). At 48 hours post dextran injection the 48-hour to 3-hour fluorescence ratio was 0.278 for morphants and 0.112 for WT (*P* = 0.002) (Figure 5F). This data is consistent with a reduction in renal excretory function in *bbs5* morphants. Fluorescent confocal microscopy confirmed pronephric duct dilatation in *bbs5* morphant embryos at 72 hpf. Interestingly, the pronephric cilia of *bbs5* morphants were less dense and had an irregular pattern in appearance (Figure 5G).

In order to study the pathogenic effects of novel human *BBS5* mutation identified in our Saudi Arabian family, we used mutant mRNA for co-injection studies to determine its ability to rescue the morphant phenotype (Figure 2F). Injection of 4 ng *bbs5* ATG MO alone showed phenotypes in 18% of fish (n = 194) whilst co-injection of *bbs5* ATG MO with WT *BBS5* mRNA in an attempt to rescue the phenotype, showed only a mild (cardiac/renal/tail) phenotype in approximately 6% of fish (n = 103). Zebrafish demonstrating complete rescue and partial rescue of the dilated pronephros, cystic pronephros and tail phenotypes using WT BBS5 mRNA are shown in Additional file 1: Figure S1. Just 3% of zebrafish co-injected with *bbs5* ATG MO and WT *BBS5* demonstrated a dilated pronephros. In these rescue experiments, there was an absence of moderate and severe phenotypes including *situs inversus* and severe tail curvature. Co-injection of *bbs5* ATG MO with mutated *BBS5* mRNA (c.966dupT) showed a (cardiac/renal/tail) phenotype in 25% of fish embryos (n = 78), including 9% severe and 7% with moderate phenotypes (Figure 2F). These experiments point to the direct pathogenicity of the c.966dupT mutation. Furthermore, histological examination of retina from morphant embryos co-injected with WT mRNA showed a recovery of the retinal layering phenotype (Figure 3G) and correction of eye size (Figure 3G). In contrast, co-injection with mutant mRNA failed to rescue the phenotype with disruption of retinal layering remaining (Figure 3H). Western blotting at 48 hours was used to confirm expression of the WT and c.966dupT mutated BBS5-NT-GFP proteins (Additional file 2: Figure S2).

### Localisation of wildtype and mutant BBS5-NT-GFP in HEK293 cells

**Figure 6:**
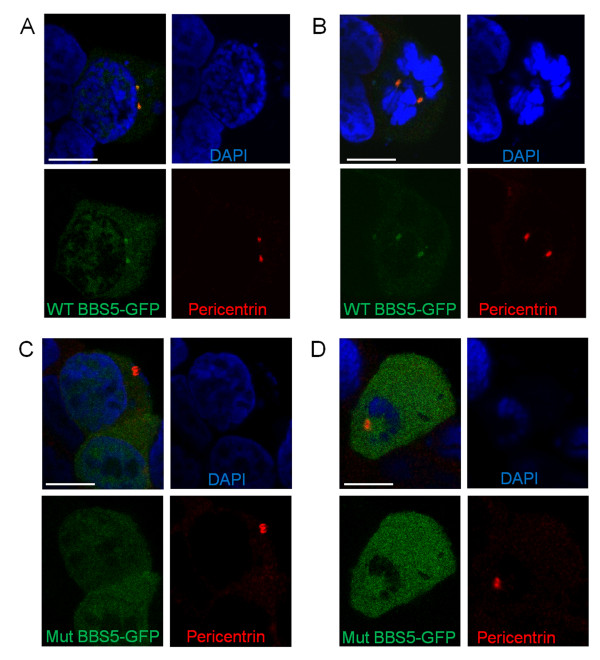
**Comparison of wildtype (WT) and mutant BBS5-NT-GFP expression and localisation in HEK293 cells.** Confocal fluorescent images (overlay, plus individual fluorescent channels respectively) from WT BBS5-NT-GFP **(A,B)** and mutant (Mut) BBS5-NT-GFP **(C,D)** are shown, together with pericentrin antibody (red) to localise centrosomes. The stain 4'-6-diamidino-2-phenylindole (DAPI) (blue) is used to stain nuclei. **(A)** WT BBS5-NT-GFP colocalises with pericentrin at centrosomes. There is some diffuse cytoplasmic and intranuclear localisation of WT BBS5-NT-GFP. **(B)** WT BBS5-NT-GFP colocalises with pericentrin at the centrosomes in a cell undergoing mitosis. **(C,D)** Mut BBS5-NT-GFP is distributed diffusely throughout the cell and fails to colocalise with the centrosomal marker pericentrin. Scale bar 10 μm.

WT and mutant BBS5-NT-GFP were transfected into HEK293 cells to assess the pathogenicity of the mutation detected (c.966dupT) at the cellular level. BBS proteins typically localize to the basal body of ciliated cells [30]. A basal body localization of WT BBS5-NT-GFP protein in HEK293 was demonstrated with colocalisation of BBS5-NT-GFP and pericentrin (Figure 6A,B). Transfection of the c.966dupT mutant BBS5-NT-GFP mRNA led to a loss of discrete basal body localisation (with loss of colocalisation with pericentrin) and an intracellular diffuse localisation (Figure 6C,D).

## Discussion

BBS is a genetically heterogeneous disorder. To date, mutations in 18 genes (*BBS1-18*) are known to cause the phenotype [14,16]. Bioinformatics analysis of these genes demonstrates that the majority of BBS genes are not genetic duplications, but are distinct genes encoding for proteins in a linked pathway [18]. In BBS, the most commonly mutated genes are *BBS1* and *BBS10*, accounting for 23% and 21% of cases respectively [31]. *BBS5* mutations are rare, with only 0.4 to 2% of cases linked to a mutation in this gene [17,31].

The intracellular role of BBS proteins has only recently begun to be understood. A breakthrough in understanding the function of BBS proteins came with the proposal by Nachury *et al*. which detailed that BBS1,2,4,5,7-9 form a functional complex named the BBSome [18]. They observed that these proteins were found in stoichiometric amounts after purification and identification with mass spectrometry, and that these proteins co-fractionate together [18]. Interestingly, the BBSome proteins are highly conserved across ciliated organisms [18]. The BBSome associates with both the ciliary membrane and with Rab8, a GTPase which is known to promote vesicular transport to the cell membrane [32]. BBS3 (or ARL6), while not a BBSome protein, is also a GTPase and thought to localise to the ciliary membrane assembly point at the base of the cilium [33], and has been shown to associate with the BBSome [34]. The BBSome localises with intraflagellar transport (IFT) particles as it travels the length of *C. reinhardtii* flagella [35] but does not seem to be a core component of the IFT complex [35]. Instead, it is proposed that the BBSome acts as an adaptor for protein cargo that is transported up the cilia by IFT [35]. These studies come together to present the current hypothesis that the BBSome, together with Rab8 and BBS3, function as adapters between vesicular bound proteins and IFT proteins to promote ciliary membrane biogenesis.

For many years, analysis of families with BBS showed evidence of a fifth locus for the disorder at position 2q31 [1,36] but it was not until a study by Li *et al*. in 2004 that the *BBS5* gene was discovered using an elegant comparative genomics method [17]. They hypothesised that unknown genes related to ciliary function will not be present in an organism without cilia, *Arabidopsis*, but present in organisms with cilia, humans and *Chlamydomonas*[17]. BBS5 is conserved and present in at least 23 orthologues including *Toxoplasma gondii*[18]. The molecular genetic diagnosis of patients with BBS is challenging because of genetic heterogeneity of BBS. We and others have shown that homozygosity mapping is a robust approach that is highly suited for genetically heterogeneous autosomal recessive disorders in populations in which consanguinity is highly prevalent. This approach of using genechip platforms of genome wide SNPs helped identify a disease locus. In the family we report, homozygosity mapping directly targeted *BBS5* as the likely cause. Phenotype-genotype correlation is typically very poor in BBS. Although we detected a mutation in *BBS5*, the phenotype we report resembles reported phenotypes from same population who had mutations in *BBS1*, *BBS3*, and *BBS4*[37].

In our family, the *BBS5* c.966dupT (p.Ala323CysfsX57) novel mutation led to a predicted elongation of the BBS protein following a change in the reading frame. To date, there are 17 previously reported mutations in *BBS5*, which include missense [17,38-43], splice site mutations [17,43] and small deletions/insertions [17,43,44]. However, to our knowledge a mutation that leads to a predicted elongation of the BBS5 protein has not previously been reported. Additional studies including RT-PCR studies and Western blotting of BBS5 protein from affected patients would be required to confirm the predicted effect of the mutation on mRNA and protein, respectively. Unfortunately this was not feasible.

In order to determine the pathogenicity of the *BBS5* mutation, we used MO injection of zebrafish embryos. The zebrafish *bbs5* shares 90.9% protein identity with human *BBS5* which allows specific modelling of human mutations. Data on a limited number (40 to 50) of *bbs5* knockout zebrafish embryos have been previously reported [26]. These embryos demonstrated abnormal Kupffer’s vesicles (KV), altered heart looping and abnormal melanosome transport. These findings show convincing evidence that *BBS5* is a ciliopathy gene with roles in multiple developmental processes.

Using mutant *BBS5* mRNA to ‘rescue’ the phenotype of *bbs5* ATG MO, we saw an enhanced number of embryos with a disease phenotype. In addition, the eye and retinal phenotype remained severe. These data suggest that the c.966dupT mutation is indeed pathogenic, although the exact mechanism is not clear. A number of possible mechanisms can be speculated. As BBS5 is part of the BBSome, the mutation may affect other BBSome proteins and the localisation and function of the BBSome. The loss of the WT C-terminal amino acids may lead to the mislocalisation of BBS5 protein and a series of C-terminal-truncating mutations could be used to identify such a centrosomal localisation motif.

A systematic approach to using zebrafish to evaluate human mutations in BBS genes has previously been reported [45] where a significant number of BBS-associated mutations were suggested to have a dominant-negative mode of action. Evidence was provided in zebrafish embryos that certain mutant mRNAs produced phenotypes significantly worse than MO alone [45]. However, it must be remembered that when using over-expression systems, there is the possibility of inducing an over-expression defect that is not relevant to the human condition.

Using zebrafish as a model for ciliopathies is well established, with reported models of Joubert syndrome [21,46,47], Meckel-Gruber syndrome, [48,49] Jeune syndrome [50] and nephronophthisis [51-53]. There have also been several other reported zebrafish models of BBS [26,29,54,55] again demonstrating retinal defects, defective melanosome transport, abnormal left-right determination with defective heart looping, abnormal KVs with defective cilia and kidney anomalies [26,29,54,55]. The establishment of left-right asymmetry in the zebrafish is secondary to an asymmetric fluid flow in KV, resulting in asymmetric gene expression across the whole of the developing embryo [56]. The abnormal cardiac looping secondary to *bbs* gene knockdown shown here and by others [26] is likely to be secondary to a cilial defect within KV and part of a generalised laterality defect.

In *bbs5* morphant zebrafish, we have demonstrated dilatation as well as pronephric and cloacal cysts. Furthermore, in the *bbs5* morphants we were able to demonstrate a reduction in renal excretory function. In zebrafish, cilia in the pronephros are motile and are important for driving fluid flow within these organs. Disruption of cilia structure or motility in the pronephros leads to fluid accumulation and cystic dilatation [57]. In *bbs5* morphants, the pronephic ducts, as well being dilated, had a reduced and irregular pattern of cilia.

Within the family we describe there were renal anomalies in two out of the three affected siblings. Renal anomalies have a reported prevalence in BBS patients of 24%, although only 52% of patients had undergone an investigative renal examination [58]. Renal anomalies may be structural changes such as renal parenchymal cysts, calyceal clubbing, foetal lobulation, dysplastic kidneys, unilateral agenesis, hydronephrosis and horseshoe kidney implicating *BBS5* in normal renal development [58]. A progressive decline in renal function in BBS patients may also be seen and may be secondary to vesicouretic reflux and obstruction leading to scarring. Renal disease is a major cause of mortality in BBS patients. Reviewing 20 BBS patients, it was revealed that all patients had either structural or functional renal abnormalities and three had established renal failure [59]. The functional defects included inability to concentrate urine and renal tubular acidification defects, implicating a dysfunctional renal collecting duct in a similar manner to nephronophthisis [60]. Chronic kidney disease progressing to established renal failure is a significant cause of morbidity and mortality in patients with BBS [8]. The findings of pronephric dilatation, together with reduced excretory function in *bbs5* morphants, implicate *bbs5* in both renal development and functional maintenance of the pronephros. We speculate that these defects may be secondary to renal ciliary dysfunction where bbs proteins coordinate ciliary proteins and ciliary signalling. Remarkably, these zebrafish morphant studies of *bbs* genes demonstrate a high degree of concordance and fit well with phenotypes reported in humans. Taken together with our data, we confirm that zebrafish are a valid model for the study of BBS genes.

At a cellular level, genes that are mutated in ciliopathies can affect ciliary signalling in different ways. This may be through changes in cilia structure, in mistargeting of signalling molecules or effecting the sensory role of primary cilia [61]. It is known that BBS5 protein is localized to basal bodies just beneath the cilia [17]. We confirm a basal body localisation for GFP-tagged BBS5, which became mislocalised upon introduction of the mutation. The (p.Ala323CysfsX57) mutation affects the C-terminal amino acids of the BBS5 protein and does not disrupt the PH domains of BBS5 [18]. Previously, it has been shown that following *BBS5* knockdown, centrin and pericentrin were still targeted to centrosomes and centriolar satellite function remained intact, however ciliogenesis was disrupted [18]. In HEK293 cells transfected with mutant BBS5, we confirm a normal expression pattern of pericentrin with complete loss of colocalisation with BBS5. This data is comparable to the *BBS4* mutant allele L327P, which failed to colocalise with γ-tubulin in post-mitotic cells [45].

## Conclusions

In conclusion we describe a consanguineous Saudi Arabian family with clinical features of BBS, together with a novel mutation in *BBS5*. Modelling this disease in zebrafish mimics the human disease, and pathogenicity of the novel *BBS5* mutation is demonstrated by the inability of mutant mRNA to rescue morphant zebrafish phenotype as well as mislocalisation of mutant BBS5 protein in renal epithelial cells.

## Abbreviations

BBS: Bardet-Biedl Syndrome; HEK: Human Embryonic Kidney; hpf: hours post fertilisation; IFT: intraflagellar transport; KV: Kupffer's vesicle; MO: Morpholino Oligonucleotide; PFA: paraformaldehyde; PH: Pleckstrin Homology; WT: Wild Type.

## Competing interests

The authors declare that they have no competing interests.

## Authors’ contributions

MHA-H carried out the molecular genetic studies, cell studies, performed zebrafish studies and drafted the manuscript. CvL, PC, RES and RJS performed zebrafish studies and drafted the manuscript. FA-F and BM helped conceive the study, recruited the patients and performed clinical studies. AMH and LE performed molecular and cell studies and drafted the manuscript. JAS conceived of the study, and participated in its design and coordination, performed zebrafish and cell studies and drafted the manuscript. All authors read and approved the final manuscript.

## Supplementary Material

Additional file 1: Figure S1 Light and fluorescence microscopy of renal cysts in bbs5 morphants and rescue with WT bbs5 mRNA. Left panels show bright-field images of 72 hpf embryos and right panels show immunofluorescence images, using claudin-Lyn-GFP embryos which express GFP throughout the pronephros (as well as forebrain and ear). (A,B) Uninjected fish (Control). (C-H) Morphological defects are seen in *bb5* morphant embryos. *bbs5* morphant embryos show pronephros dilatation and cyst formation which is subtle on light microscopy (black arrows) but more easily identified under fluorescence microscopy (white arrows). (I-N) Morphant phenotypes of tail abnormalities, pronephric duct dilatation /cysts are (I-L) partially and (M,N) fully rescued by co-injection with WT bbs5 mRNA. Click here for file

Additional file 2: Figure S2 Expression of WT and mutant BBS5-NT-GFP. Western blotting confirming protein expression of the predicted size (arrowed) for WT and mutant BBS-NT-GFP (predicted molecular weight 45 kDa BBS5 + 27 kDa GFP = 72 kDa). Click here for file
