# Supplementary figures and images for: Functional modelling of a novel mutation in BBS5
Source: Cilia. 2014 Feb 21;3:3. doi: 10.1186/2046-2530-3-3 (PMC3931281; doi:10.1186/2046-2530-3-3)

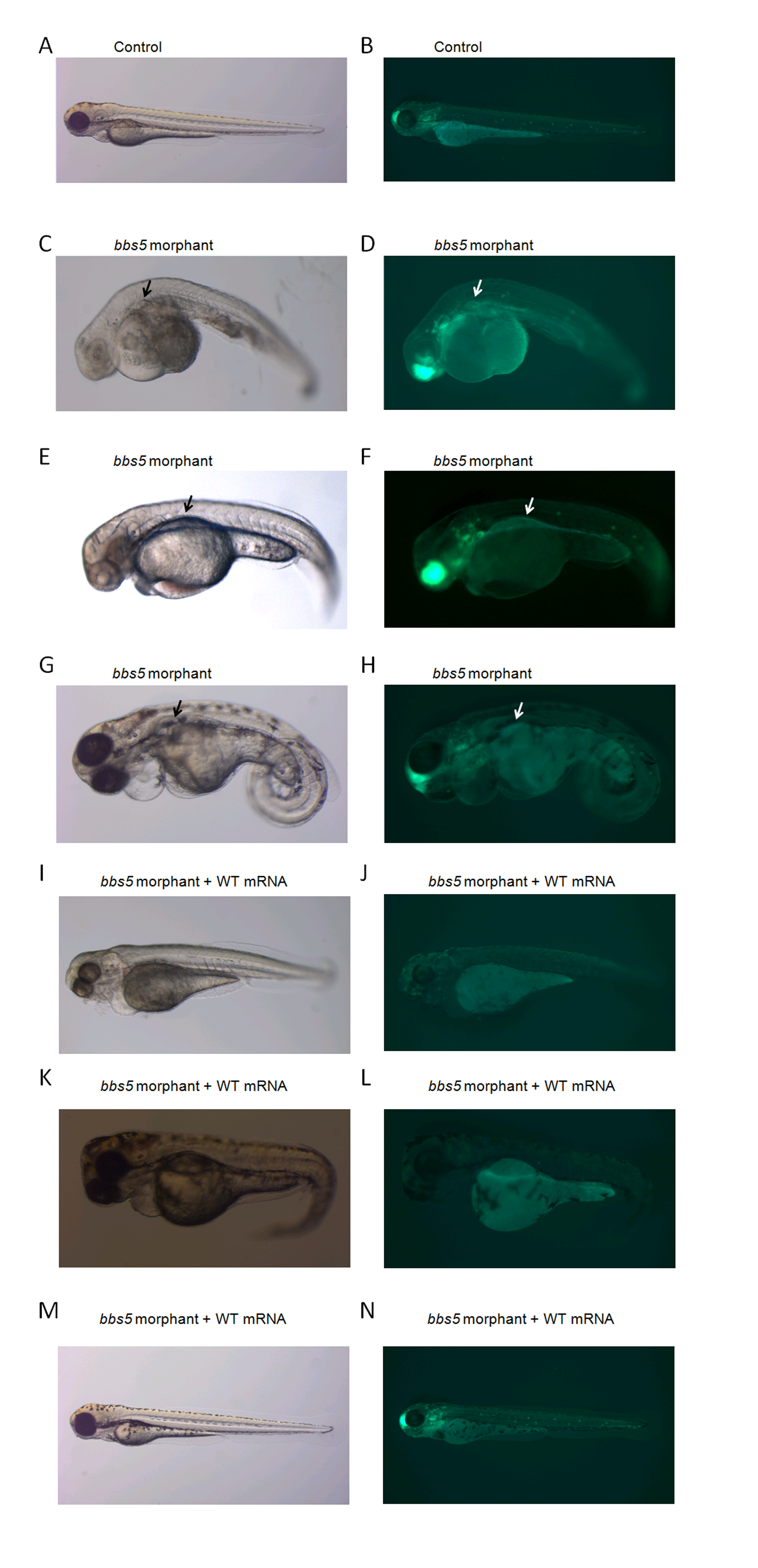

Supplement: Additional file 1: Figure S1 — Light and fluorescence microscopy of renal cysts in bbs5 morphants and rescue with WT bbs5 mRNA. Left panels show bright-field images of 72 hpf embryos and right panels show immunofluorescence images, using claudin-Lyn-GFP embryos which express GFP throughout the pronephros (as well as forebrain and ear). (A,B) Uninjected fish (Control). (C-H) Morphological defects are seen in bb5 morphant embryos. bbs5 morphant embryos show pronephros dilatation and cyst formation which is subtle on light microscopy (black arrows) but more easily identified under fluorescence microscopy (white arrows). (I-N) Morphant phenotypes of tail abnormalities, pronephric duct dilatation /cysts are (I-L) partially and (M,N) fully rescued by co-injection with WT bbs5 mRNA. [file 2046-2530-3-3-S1.tiff]

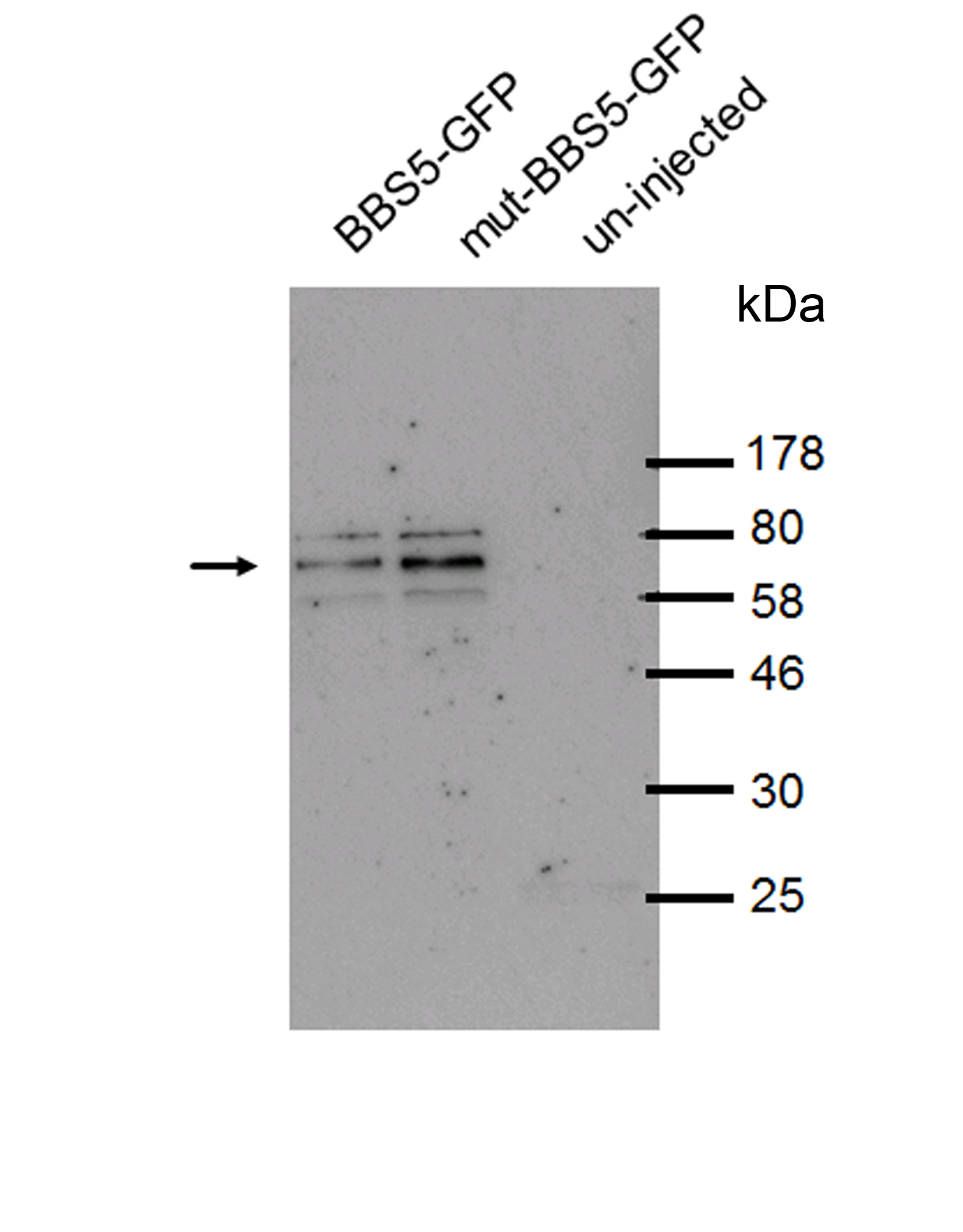

Supplement: Additional file 2: Figure S2 — Expression of WT and mutant BBS5-NT-GFP. Western blotting confirming protein expression of the predicted size (arrowed) for WT and mutant BBS-NT-GFP (predicted molecular weight 45 kDa BBS5 + 27 kDa GFP = 72 kDa). [file 2046-2530-3-3-S2.tiff]
